# Urinary Cystatin C Has Prognostic Value in Peripheral Artery Disease

**DOI:** 10.3390/biom12070860

**Published:** 2022-06-21

**Authors:** Ben Li, Abdelrahman Zamzam, Muzammil H. Syed, Niousha Jahanpour, Shubha Jain, Rawand Abdin, Mohammad Qadura

**Affiliations:** 1 Division of Vascular Surgery, St. Michael’s Hospital, Unity Health Toronto, University of Toronto, Toronto, ON M5B 1W8, Canada; benx.li@mail.utoronto.ca (B.L.); abdelrahman.zamzam@unityhealth.to (A.Z.); muzammil.syed@mail.utoronto.ca (M.H.S.); niousha.jahanpour@gmail.com (N.J.); jains@ucalgary.ca (S.J.); 2 Department of Medicine, McMaster University, Hamilton, ON L8S 4K1, Canada; rawand.abdin@medportal.ca; 3 Department of Surgery, University of Toronto, Toronto, ON M5S 1A1, Canada; 4 Keenan Research Centre for Biomedical Science, Li Ka Shing Knowledge Institute, St. Michael’s Hospital, Unity Health Toronto, University of Toronto, Toronto, ON M5B 1W8, Canada

**Keywords:** Cystatin C, peripheral arterial disease, major adverse limb events

## Abstract

Despite its association with adverse outcomes, peripheral artery disease (PAD) remains undertreated. Cystatin C is elevated in patients with renal disease and may be a marker of cardiovascular disease. We examined the prognostic ability of urinary Cystatin C (uCystatinC) in predicting adverse PAD-related events. In this prospective case-control study, urine samples were collected from patients with PAD (*n* = 121) and without PAD (*n* = 77). The cohort was followed for 2 years. uCystatinC was normalized to urinary creatinine (uCr) (uCystatinC/uCr; μg/g). The primary outcome was major adverse limb event (MALE; composite of vascular intervention (open or endovascular) or major limb amputation). The secondary outcome was worsening PAD status (drop in ABI ≥ 0.15). Multivariable Cox regression and Kaplan–Meier analyses were performed to assess the prognostic value of uCystatinC/uCr with regards to predicting MALE and worsening PAD status. Our analysis demonstrated that patients with PAD had significantly higher median [IQR] uCystatinC/uCr levels (24.9 μg/g [14.2–32.9] vs. 20.9 μg/g [11.1–27.8], *p* = 0.018). Worsening PAD status and MALE were observed in 39 (20%) and 34 (17%) patients, respectively. uCystatinC/uCr predicted worsening PAD status with a hazard ratio (HR) of 1.78 (95% CI 1.12–2.83, *p* = 0.015), which persisted after controlling for baseline demographic and clinical characteristics (adjusted HR 1.79 [95% CI 1.11–2.87], *p* = 0.017). Patients with high uCystatinC/uCr had a lower 2-year freedom from MALE (77% vs. 89%, *p* = 0.025) and worsening PAD status (63% vs. 87%, *p* = 0.001). Based on these data, higher uCystatinC/uCr levels are associated with adverse PAD-related events and have prognostic value in risk-stratifying individuals for further diagnostic vascular evaluation or aggressive medical management.

## 1. Introduction

Peripheral artery disease (PAD) involves atherosclerosis of the lower extremity arteries and affects over 200 million people worldwide [1]. Despite its association with poor cardiovascular outcomes, PAD remains undertreated [2]. As a result, patients with PAD usually have worse long-term prognosis compared to patients with coronary artery disease (CAD) [3]. This is partly because there lacks a validated risk stratification and prognostication biomarker for PAD [4]. The commonly used ankle-brachial index (ABI) is limited by operator dependence, erroneous interpretation, and unreliability in patients with diabetes [5,6]. Most primary care providers do not perform ABIs routinely due to time constraints, unavailability of skilled personnel, and complexity of result interpretation. Clinicians view blood or urine tests as preferable to ABIs and would enhance simplicity and efficiency for PAD management [7]. Therefore, the identification of a prognostication biomarker for PAD may improve cardiovascular risk stratification through initiation of aggressive medical management, close ambulatory follow-up, and vascular interventions.

In addition, ABIs are poor predictors of PAD-related complications and medical management of PAD remains poor, partly due to the inability to routinely identify high-risk patients [8,9]. The Cardiovascular Outcomes for People Using Anticoagulation Strategies (COMPASS) trial demonstrated a significant reduction in major adverse cardiovascular events for patients with atherosclerotic vascular disease taking low-dose rivaroxaban (2.5 mg oral twice daily) in addition in ASA [10]. Therefore, identifying high-risk PAD patients using a novel prognostic biomarker can allow clinicians to better select patients for intensive medical therapy, including the COMPASS trial low-dose rivaroxaban. 

Cystatin C is a serum protein produced by nucleated cells that has traditionally been viewed as a biomarker for renal function [11]. However, recent studies have demonstrated that Cystatin C is expressed in atherosclerotic lesions and may predict adverse cardiovascular outcomes in patients with CAD and PAD [12,13,14]. Since serum Cystatin C is filtered by the kidneys, its levels can be measured in urine (uCystatinC). Given that urine tests can be obtained less invasively than blood tests, the identification of a urinary biomarker may allow for more widespread application in the prognosis and management of PAD. In this study, we assessed the prognostic value of uCystatinC in PAD.

## 2. Materials and Methods

### 2.1. Ethics Approval

This study was approved by the Unity Health Toronto Research Ethics Board. Written informed consent was obtained from all patients. Methods were carried out in accordance with the World Medical Association Declaration of Helsinki [15].

### 2.2. Patient Recruitment and Assessment

A single-center prospective case-control study was conducted. Consecutive patients with and without PAD presenting to vascular surgery ambulatory clinics at St. Michael’s Hospital, University of Toronto between August 2018 and August 2019 were recruited. Details regarding the patients’ clinical information, physical exam, lower limb arterial ultrasound, and ankle-brachial index (ABI) were recorded. The diagnosis of peripheral artery disease (PAD) was confirmed during the initial patient encounter by clinical assessment and imaging. As previously described by our work [16,17] and the Society for Vascular Surgery guidelines [18], PAD was defined as ABI < 0.9, in addition to a lack of posterior tibial and/or dorsalis pulses in at least one leg, with or without claudication [19]. The non-PAD group was identified as having a normal arterial ultrasound, an ABI ≥ 0.9, presence of palpable distal pulses, and no clinical history of claudication [19]. In situations where the ABI could not be accurately determined due to non-compressible tibial vessels, toe-brachial index (TBI) measurements were performed [19]. Patients with TBI < 0.67 were characterized as having PAD, whereas controls had a TBI of ≥0.67 [19]. Patients with a history of chronic kidney disease (stages 3–5 as per Kidney Disease Outcomes Quality Initiative 2002 guidelines—defined as having an estimated glomerular filtration rate of less than 60 mL/min/1.73 m^2^) were excluded [20]. We also excluded patients with a diagnosis of sepsis in the past 3 months or malignancy. Patients with chronic kidney disease, sepsis, and malignancy were excluded to reduce confounding as these factors may increase Cystatin C levels.

### 2.3. Baseline Measurements and Sample Collection

We recorded baseline demographics, history of cardiovascular diseases, cardiovascular risk factors (hypertension, dyslipidemia, and diabetes), and smoking status as described previously [16]. Mid-stream urine samples were obtained during the initial assessment, aliquoted, and stored at −80 °C prior to analysis. Urine samples were thawed slowly on ice prior to analysis.

### 2.4. Urinary Cystatin C Multiplex Assay

The concentration of urinary Cystatin C was determined by a multiplexed suspension array system using MILLIPLEX^®^ Human Kidney Injury Magnetic Bead Panel 2 (EMD-Millipore; Billerica, MA, USA) [21]. The manufacturer’s protocol and recommendations were followed for the multiplex bead assays [21]. The sample intra-assay coefficient of variability (CV) was <10% while the inter-assay CV was 15%, which meets the threshold for statistical acceptability [22]. Prior to any sample analysis, Fluidics Verification and Calibration bead kits (Luminex Corp, Austin, TX, USA) [23] were used to calibrate the MagPix analyzer (Luminex Corp; Austin, TX, USA) [24]. At least 50 beads for Cystatin C were acquired using Luminex xPonent software and analyzed using Milliplex Analyst software (v.5.1; EMD-Millipore) [25].

### 2.5. Measurement of Urinary Creatinine and Normalization of Cystatin C

Urine creatinine (uCr) levels were measured at the Core Laboratory at St. Michael’s Hospital using the Beckman Coulter AU680 laboratory analyzer (Beckman Coulter; Pasadena, CA, USA) [26]. The Cystatin C concentrations were normalized to uCr to adjust for urinary concentration errors and differences in hydration status while single-spot urine samples were used to achieve normalized uCystatinC/uCr (μg/g) as we have previously described [16,17].

### 2.6. Outcomes

The primary endpoint was major adverse limb events (MALEs), defined as the requirement for vascular intervention (open or endovascular revascularization) or major amputation (above the level of the ankle) [27]. The secondary endpoint was worsening PAD status, defined as a change in ABI ≥ 0.15 from baseline, which has been demonstrated to be clinically significant [28,29,30]. 

### 2.7. Follow-Up

Patient follow-up was performed at 12 and 24 months. During these follow-up visits, ABI values, PAD-related interventions, and any changes made to concomitant treatment were recorded. PAD-related hospitalization and emergency room visits were also recorded.

### 2.8. Statistical Analysis

Demographic and clinical characteristics were expressed as the mean and standard deviations (SD) or number and proportions (%). Continuous data were compared using Student’s *t*-test if conforming to a normal distribution; otherwise, the Mann–Whitney U test was used. The Chi-square test was used for categorical variable comparisons. Pearson’s correlation coefficient was used to measure the statistical association between continuous variables. Normalized uCystatinC/uCr levels were not normally distributed, as determined by the Kolmogorov–Smirnov test, and were logarithmically transformed before survival analysis and expressed as medians with interquartile ranges (IQRs).

To find the relationship between uCystatinC/uCr and PAD severity based on ABI, we measured uCystatinC/uCr levels in subgroups of patients with no PAD (ABI ≥ 0.90), mild PAD (ABI 0.89–0.75), moderate PAD (ABI 0.74–0.50), and severe PAD (ABI < 0.50) as defined by the European Society for Vascular Medicine (ESVM) guidelines. We also performed subgroup analysis to assess the impact of baseline ABI on outcomes.

Event rates at 24 months were calculated for worsening PAD status, MALE, vascular intervention, and major amputation. A Cox proportional hazard analysis was performed to determine the uCystatinC/uCr predictability for primary and secondary outcomes. Baseline demographic and clinical variables (age (in years), sex (male vs. female), hypertension (yes vs. no), dyslipidemia (yes vs. no), smoking (yes vs. no), diabetes (yes vs. no), and history of CAD (yes vs. no)) were entered into the multivariate analysis. For the Kaplan–Meier analysis, the median uCystatinC/uCr level of the whole cohort was used as the cutoff point to risk-stratify patients into low and high uCystatinC/uCr. Freedom from events was computed and differences between groups were assessed using the log-rank test. Statistical significance was established at *p* < 0.05 (2-sided). SPSS software, version 23 (SPSS Inc., Chicago, IL, USA) was used for statistical analysis [31]. 

## 3. Results

### 3.1. Patient Characteristics

Overall, 198 patients (121 with PAD and 77 without PAD) were included in our study. Patients with PAD were older (67 [SD 10] vs. 63 [SD 13], *p* = 0.008) and more likely to have comorbidities, including hypertension (74% vs. 60%, *p* = 0.042), dyslipidemia (85% vs. 57%, *p* = 0.001), diabetes (31% vs. 16%, *p* = 0.017), and CAD (37% vs. 18%, *p* = 0.004). The mean ABI was lower in PAD patients (0.64 vs. 0.97, *p* = 0.001) (Table 1).

Patients with PAD were more likely to receive a statin (86% vs. 58%, *p* = 0.001), angiotensin-converting enzyme inhibitors (ACE-Is) or angiotensin II receptor blockers (ARBs) (63% vs. 35%, *p* = 0.001), and aspirin (64% vs. 47%, *p* = 0.019) compared to patients without PAD. There were no differences in the proportion of patients receiving other antiplatelets or anticoagulants

Patients with more severe PAD based on ABI had higher levels of median (IQR) uCystatinC/uCr levels (severe PAD [28.5 (14.5–35.1)], moderate PAD [24.0 (14.1–36.8)], mild PAD [23.5 (10.9–28.0)], and non-PAD [20.9 (11.1–27.9)]). Patients with any PAD severity were more likely to develop adverse events than those without PAD. However, there was no significant association between PAD severity and adverse events. This may be related to the fact that in this prospective study, patients were being followed closely by vascular surgeons and revascularized in a timely manner to reduce the risk of adverse events, particularly for patients with moderate and severe PAD (Supplementary Table S1).

### 3.2. Association between uCystatinC/uCr and PAD-Related Adverse Events

uCystatinC/uCr levels were not associated with age, sex, hypertension, dyslipidemia, diabetes, smoking, history of congestive heart failure, or history of coronary artery disease. Significant differences were seen between the median uCystatinC/uCr levels in patients with PAD compared to patients without PAD (24.9 [14.2–32.9] vs. 20.9 [11.1–27.8], *p* = 0.018) and patients with diabetes compared to patients without diabetes (26.0 [19.7–37.1] vs. 21.7 [11.1–29.2], *p* = 0.006) (Table 2).

In total, 39/198 (20%) patients had worsening PAD status over the 2-year follow up period (26% [PAD] vs. 9% [non-PAD], *p* = 0.005). We also observed that 34/198 (17%) developed MALE, 30/198 (15%) required vascular intervention, and 5/198 (3%) had major amputation (Table 3). There was a significant association between every one unit increase in log (uCystatin/uCr) and worsening PAD status (unadjusted HR 1.78 [95% CI 1.12–2.83], *p* = 0.015; adjusted HR 1.79 [95% CI 1.11–2.87], *p* = 0.017) (Table 4). 

### 3.3. Risk-Stratification Based on uCystatinC/uCr

**Figure 1:**
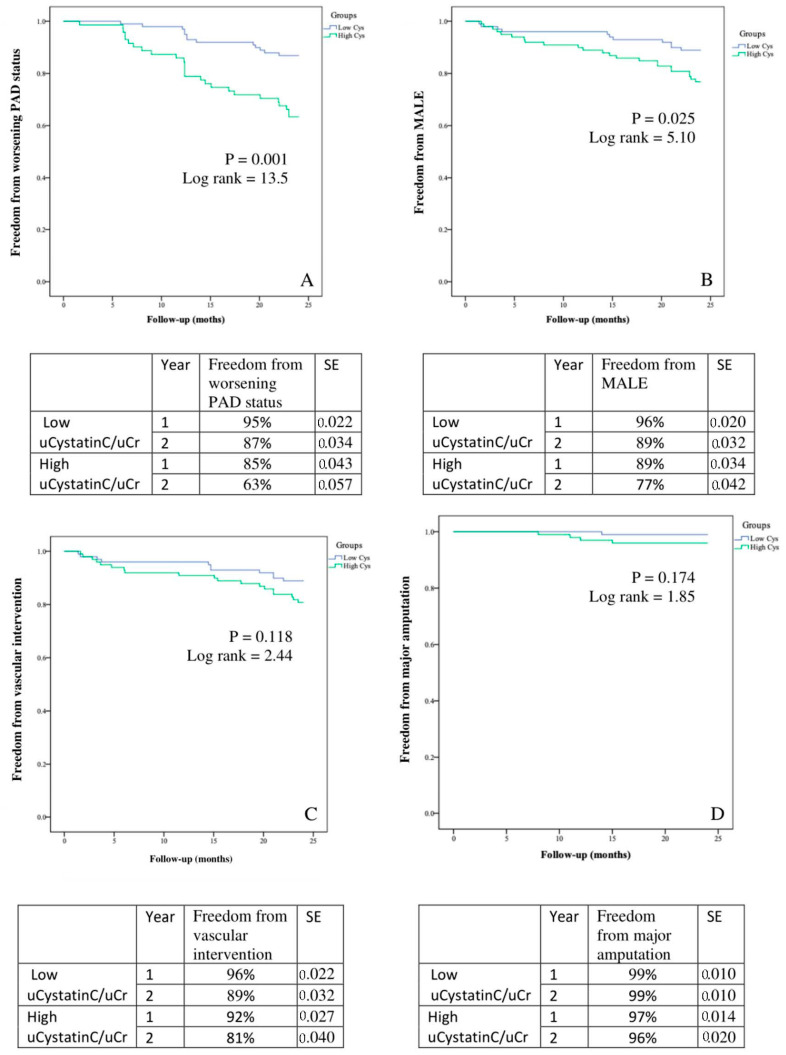
Kaplan–Meier analysis of event-free survival rates in patients with low vs. high urinary Cystatin C normalized to urinary creatinine (uCystatinC/uCr) for (**A**) worsening PAD (ankle-brachial index drop ≥0.15), (**B**) MALE (major adverse limb event), (**C**) vascular intervention, and (**D**) major amputation. SE = standard error.

Patients were stratified into low vs. high uCystatinC/uCr based on the median uCystatinC/uCr value of 23.2 μg/g. Those with high uCystatinC/uCr were more likely to have PAD (70% vs. 52%, *p* = 0.04) and be a current or past smoker (87% vs. 74%, *p* = 0.006). Patients with high uCystatinC/uCr were also more likely to have worsening PAD status (26% vs. 13%, *p* = 0.020) and MALE (23% vs. 11%, *p* = 0.024) (Table 5). Based on Kaplan–Meier analysis over a 2-year period, patients with high uCystatinC/uCr had a lower freedom from MALE (77% vs. 89%, *p* = 0.032) and worsening PAD status (63% vs. 87%, *p* = 0.001) (Figure 1). 

## 4. Discussion

### 4.1. Summary of Findings

Our single-center prospective case-control study demonstrates that uCystatinC/uCr has prognostic value in PAD. We identified a cohort of 198 patients with and without PAD and demonstrated that uCystatinC/uCr was elevated in the PAD group. Furthermore, higher levels of uCystatinC/uCr predicted worsening PAD status. We then risk-stratified our cohort into low and high uCystatinC/uCr and found that patients with uCystatinC/uCr were more likely to be diagnosed with PAD. Finally, we showed that patients with high uCystatinC/uCr were more likely to progress in their PAD disease state and develop MALE over a 2-year period. 

### 4.2. Comparison to Existing Literature

Cystatin C is a cysteine-protease inhibitor and low-molecular-weight protein produced by nucleated cells [32]. Traditionally, this protein has been used to estimate the glomerular filtration rate, particularly in acute kidney injury, due to its high sensitivity to rapid changes in renal function [33,34]. More recently, Cystatin C has been found to be expressed in atherosclerotic lesions and play an important role in vascular remodeling [13]. Several studies have demonstrated that serum Cystatin C can predict cardiovascular mortality and morbidity in patients with CAD and PAD [35,36,37]. Urbonaviciene et al. (2011) showed that PAD patients with high serum cystatin C levels had higher all-cause mortality (adjusted HR 2.99) and cardiovascular mortality (adjusted HR 4.36) [35]. Qing et al. (2012) demonstrated that serum cystatin C was elevated in patients with asymptomatic CAD (OR 1.33) [38]. Kim et al. (2015) showed that serum cystatin C predicted the incidence of contrast-induced nephropathy in PAD patients undergoing endovascular intervention, with an area under the receiver operating characteristic curve (AUROC) of 0.76 [37]. In our prospective study, we similarly demonstrated that urinary Cystatin C predicted PAD-related adverse events, including worsening PAD status and MALE outcomes. Given that urine tests can be obtained less invasively than blood tests, the identification of this urinary biomarker for PAD may allow for more widespread application in the prognosis and management of PAD. 

### 4.3. Explanation of Findings

There are several potential explanations for our findings. First, we demonstrated that PAD patients are more likely to have CAD due to a greater burden of related cardiovascular risk factors, including hypertension, dyslipidemia, and diabetes. This corroborates previous evidence demonstrating that individuals with PAD often have systemic disease, with the majority of PAD patients being diagnosed with CAD [39]. Therefore, they are at a significantly higher risk of cardiovascular death compared to patients without PAD [40]. This highlights the importance of developing a widely applicable screening and prognostic tool for PAD. Second, we found that uCystatinC/uCr levels were higher in PAD patients. A potential mechanism is that inflammatory cytokines associated with atherosclerosis may alter the relationship between cysteine protease and their endogenous inhibitor cystatin C [41,42,43]. Measurement of the levels of cystatin C may characterize this imbalance and provide insight into the severity and prognosis of atherosclerosis [41,42,43]. Overall, this provides a valuable biomarker that can be measured non-invasively in patients to screen for PAD. Third, patients with high uCystatinC were more likely to develop MALE and have worsening PAD over a 2-year period. This suggests that uCystatinC/uCr has prognostic value in PAD. Importantly, we demonstrated that high uCystatinC/uCr was associated with worse PAD prognosis independent of CAD. In particular, there was no association between uCystatinC/uCr and history of CAD in our study. Furthermore, patients with chronic kidney disease, sepsis, and malignancy were excluded, which significantly reduced the risk of confounding. Therefore, this biomarker has the potential to risk-stratify patients and guide clinicians with regards to the aggressiveness of medical management and intensity of follow-up, and allocation of health care resources. There are significant gaps in the medical management of PAD [9], which may be addressed through a prognostic biomarker that can improve the identification of high-risk patients that require more intensive therapy, such as low-dose rivaroxaban, which has been shown to provide cardiovascular mortality benefit in the COMPASS trial [10]. Finally, there was likely no difference in 2-year major amputation between groups due to the low incidence of this event. Furthermore, patients were followed closely in this prospective study and likely underwent early revascularization to prevent limb loss. uCystatinC/uCr likely predicted 2-year MALE but not vascular intervention or major amputation because of the higher event rate of the composite outcome.

### 4.4. Limitations

This study has several limitations. First, this was an observational study and there were differences in the baseline characteristics between groups. However, our analysis adjusted for important baseline characteristics, including age, sex, hypertension, dyslipidemia, diabetes, smoking, and CAD. The findings remained significant after controlling for confounders. Second, 2-year outcomes were reported, and longer follow-up is needed to better understand the prognostic value of uCystatinC/uCr given that PAD is generally a long-standing disease. Third, we excluded patients with chronic kidney disease, malignancy, and sepsis within the past 3 months. Therefore, the results may not apply to all PAD patients and further investigation of uCystatin C is needed to better understand how the biomarker can be effectively implemented as a prognostic tool in clinical practice. Fourth, given our study design, we could not compare the prognostic value of uCystatinC/uCr to that of either eGFR or serum Cr levels in our cohort as patients with stage 3–5 chronic kidney disease were excluded to reduce risk of confounding for this urinary biomarker. Lastly, the relatively small sample size and short follow-up period may serve as a source of potential bias. Future studies with larger sample sizes and an increased follow-up period are warranted.

## 5. Conclusions

Our study demonstrates two important findings. First, elevated uCystatinC/uCr is associated with PAD. Second, patients with high uCystatinC/uCr are more likely to develop MALE and have worsening PAD even after adjusting for CAD history. These results suggest that uCystatinC/uCr has good prognostic value in PAD. Measurement of uCystatinC/uCr may improve risk stratification and identify patients at high risk of developing PAD-related complications. These patients may then benefit from additional diagnostic evaluation, close follow-up, and aggressive medical management, such as prescription of low-dose rivaroxaban. Urine samples are easily collected and analyzed in the primary care setting, and, therefore, this biomarker has potential for widespread clinical implementation as a PAD screening and prognostic tool. Larger studies and clinical trials should be performed to confirm these findings.

## Figures and Tables

**Table 1:** Demographic and clinical characteristics of patients with and without peripheral artery disease.

	Overall (*n* = 198)	Non-PAD (*n* = 77)	PAD (*n* = 121)	*p*
Mean (SD) ^‡^				
**Ankle-brachial index**	0.83 (0.26)	0.97 (0.21)	0.64 (0.20)	***0.001***
**Age, years**	66 (11)	63 (13)	67 (10)	***0.008***
**N (%) ^¶^**				
**Sex, male**	128 (65)	50 (65)	78 (65)	0.946
**Hypertension**	135 (68)	46 (60)	89 (74)	***0.042***
**Dyslipidemia**	147 (74)	44 (57)	103 (85)	***0.001***
**Diabetes**	49 (25)	12 (16)	37 (31)	***0.017***
**Smoking, current + past**	161 (81)	54 (70)	107 (88)	***0.001***
**History of congestive heart failure**	5 (3)	1 (1)	4 (3)	0.395
**History of coronary artery disease**	59 (30)	14 (18)	45 (37)	***0.004***
**Medications** **N (%) ^¶^**				
**Statins**	149 (75)	45 (58)	104 (86)	**0.001**
**ACEi/ARB**	102 (52)	27 (35)	75 (63)	**0.001**
**Βeta Blockers**	52 (27)	17 (22)	35 (29)	0.26
**Insulin**	13 (7)	5 (7)	8 (7)	0.97
**Oral Hypoglycemic**	32 (16)	12 (16)	20 (17)	0.86
**Aspirin**	113 (57)	36 (47)	77 (64)	**0.019**
**Other antiplatelets (Plavix, Pradaxa, Ticagrelor)**	41 (21)	11 (14)	30 (25)	0.08
**2.5 mg dose rivaroxaban**	4 (2)	0 (0)	4 (3)	0.15

ABI = ankle-brachial index. ACEi/ARB = angiotensin-converting enzyme inhibitor/angiotensin receptor blocker. ^‡^ Compared using Student’s t-test or Mann-Whitney U test. **^¶^** Compared using the Chi-square test. Abbreviations: PAD = peripheral artery disease, uCystatinC/uCr = urinary Cystatin C normalized to urinary creatinine.

**Table 2:** Association between uCystatinC/uCr levels and demographic/clinical characteristics.

	Mean (SD)	Pearson Correlation	*p*
**Age**	66 (11)	0.017	0.817
	**uCystatinC/uCr levels (Median [IQR])**		***p***
**PAD**	**Yes**	**No**	
	24.9 (14.2–32.9)	20.9 (11.1–27.8)	***0.018***
**Sex**	**Male**	**Female**	
	22.8 (14.1–29.0)	23.9 (12.4–32.9)	0.811
**Hypertension**	**Yes**	**No**	
	22.6 (13.0–30.4)	24.6 (14.9–29.9)	0.470
**Dyslipidemia**	**Yes**	**No**	
	23.1 (13.6–29.6)	23.2 (15.7–30.8)	0.997
**Diabetes**	**Yes**	**No**	
	26.0 (19.7–37.1)	21.7 (11.1–29.2)	***0.006***
**Smoking, current + past**	**Yes**	**No**	
	24.2 (14.1–30.9)	18.7 (11.1–25.9)	0.051
**History of congestive heart failure**	**Yes**	**No**	
	19.3 (10.4–37.0)	23.4 (13.7–29.9)	0.920
**History of coronary artery disease**	**Yes**	**No**	
	24.2 (13.1–33.9)	22.3 (13.9–29.4)	0.307

Abbreviations: uCystatinC/uCr = urinary Cystatin C normalized to urinary creatinine, PAD = peripheral artery disease.

**Table 3:** Adverse events in patients with and without peripheral artery disease during the 2-year follow-up.

	Overall (*n* = 198)	Non-PAD (*n* = 77)	PAD (*n* = 121)	*p* *
**Worsening PAD status (change in ABI ≥ 0.15)**	39 (20)	7 (9)	32 (26)	***0.005***
**MALE**	34 (17)	0 (0)	34 (28)	***0.001***
**Vascular intervention**	30 (15)	0 (0)	30 (25)	***0.001***
**Major amputation**	5 (3)	0 (0)	5 (4)	0.062

Results are presented as N (%). * Compared using the Chi-square test. Abbreviations: PAD = peripheral artery disease, ABI = ankle-brachial index, MALE = major adverse limb event; composite of vascular intervention and major amputation.

**Table 4:** Multivariable Cox regression models for associations between every one unit increase in log (uCystatinC/uCr) and PAD-related adverse events.

	Unadjusted HR (95% CI)	*p*	Adjusted HR (95% CI) ^‡^	*p*
**Worsening PAD status (change in ABI ≥ 0.15)**	1.78 (1.12–2.83)	***0.015***	1.79 (1.11–2.87)	***0.017***
**MALE**	1.20 (0.87–1.66)	0.258	1.09 (0.81–1.46)	0.562
**Vascular intervention**	1.08 (0.846–1.38)	0.533	1.02 (0.802–1.30)	0.868
**Major amputation**	1.01 (0.36–1.95)	0.893	1.01 (0.39–1.85)	0.754

^‡^ Adjusted for age, sex, hypertension, dyslipidemia, smoking, diabetes, and history of CAD. Abbreviations: PAD = peripheral artery disease, ABI = ankle-brachial index, MALE = major adverse limb event; composite of vascular intervention and major amputation.

**Table 5:** Baseline demographic and clinical characteristics of patients with low and high uCystatinC/uCr.

	Low uCystatinC/uCr (*n* = 99)	High uCystatinC/uCr (*n* = 99)	*p*
Mean (SD) ^‡^			
**Age, years**	67 (10)	66 (11)	0.313
**N (%) ^¶^**			
**PAD**	51 (52)	69 (70)	***0.04***
**Sex, male**	65 (66)	63 (64)	0.766
**Hypertension**	70 (71)	65 (66)	0.446
**Dyslipidemia**	74 (75)	73 (74)	0.871
**Diabetes**	20 (20)	30 (30)	0.070
**Smoking, current + past**	73 (74)	88 (87)	***0.006***
**History of coronary artery disease**	26 (26)	33 (33)	0.418
**Event rate at 2 years N (%) ^¶^**			
**Worsening PAD status (change in ABI ≥ 0.15)**	13 (13)	26 (26)	***0.020***
**MALE**	11 (11)	23 (23)	***0.024***
**Vascular intervention**	11 (11)	19 (19)	0.113
**Major limb amputation**	1 (1)	4 (4)	0.174

Low uCystatinC/uCr: ≤23.2 μg/g. High uCystatinC/uCr: >23.2 μg/g. ^‡^ Compared using Student’s *t*-test. **^¶^** Compared using the Chi-square test. Abbreviations: PAD = peripheral artery disease, ABI = ankle-brachial index, MALE = major adverse limb event; composite of vascular intervention and major amputation).

## Data Availability

The data presented in this study are available in the manuscript itself.

## Supplementary Materials

The following supporting information can be downloaded at: https://www.mdpi.com/article/10.3390/biom12070860/s1, Table S1:Levels of normalized uCystatinC/uCr among non-PAD controls (*n* = 77) and PAD patients stratified based on their ABI values (mild PAD: 0.89–0.75, *n* = 34; moderate PAD: 0.74–0.50, *n* = 57; severe PAD: <0.50, *n* = 30) as per the European Society for Vascular Medicine (ESVM) guidelines on peripheral arterial disease (Pubmed ID 31789115).
