# Supplementary material for: Urinary Cystatin C Has Prognostic Value in Peripheral Artery Disease
Source: Biomolecules. 2022 Jun 21;12(7):860. doi: 10.3390/biom12070860 (PMC9313414; doi:10.3390/biom12070860)
Supplement: Supplementary file 1 [file biomolecules-12-00860-s001.zip › biomolecules-1761921-supplementary.pdf]

biomolecules

Supplementary section:

Table S1. Levels of normalized uCystatinC/uCr among non-PAD controls ($n = 77$) and PAD patients stratified based on their ABI values (mild PAD: 0.89–0.75, $n = 34$; moderate PAD: 0.74–0.50, $n = 57$; severe PAD: < 0.50, $n = 30$) as per the European Society for Vascular Medicine (ESVM) guidelines on peripheral arterial disease (pubmed ID 31789115).

	Non-PAD ($n = 77$)	Mild ($n = 34$)	Moderate ($n = 57$)	Severe ($n = 30$)	p Value
ABI Range	> 0.90	0.89–0.75	0.74–0.50	< 0.50	
Median (IQR) ‡					
Normalized uCystatinC/uCr (µg/g)	20.9 (11.1–27.9)	23.5 (10.9–28.0)	24.0 (14.1–36.8)	28.5 (14.5–35.1)	***0.036***
**Event rate at 2 years N (%)¶**					
Worsening PAD status (change in ABI ≥ 0.15)	7 (9)	17 (50)	11 (19)	4 (13)	***0.001***
MALE	0 (0)	18 (53)	8 (14)	8 (27)	***0.001***
Vascular intervention	0 (0)	15 (44)	7 (12)	8 (27)	***0.001***
Major amputation	0 (0)	2 (6)	0 (0)	3 (10)	***0.008***

‡ Compared using student's *t*-test. ¶ Compared using chi-square test. Abbreviations: ABI (ankle-brachial index), PAD (peripheral artery disease), Cr Creatinine, MALE (major adverse limb event; composite of vascular intervention and major amputation).
